# Next Generation Plasma Proteomics Identifies High-Precision Biomarker Candidates for Ovarian Cancer

**DOI:** 10.3390/cancers14071757

**Published:** 2022-03-30

**Authors:** Ulf Gyllensten, Julia Hedlund-Lindberg, Johanna Svensson, Johanna Manninen, Torbjörn Öst, Jon Ramsell, Matilda Åslin, Emma Ivansson, Marta Lomnytska, Maria Lycke, Tomas Axelsson, Ulrika Liljedahl, Jessica Nordlund, Per-Henrik Edqvist, Tobias Sjöblom, Mathias Uhlén, Karin Stålberg, Karin Sundfeldt, Mikael Åberg, Stefan Enroth

**Affiliations:** 1 Department of Immunology, Genetics, and Pathology, Biomedical Center, SciLifeLab Uppsala, Uppsala University, SE-75108 Uppsala, Sweden; ulf.gyllensten@igp.uu.se (U.G.); julia.hedlund_lindberg@igp.uu.se (J.H.-L.); emma.ivansson@igp.uu.se (E.I.); per-henrik.edqvist@igp.uu.se (P.-H.E.); tobias.sjoblom@igp.uu.se (T.S.); 2 Stellenbosch Institute for Advanced Study (STIAS), Marais Rd., Mostertsdrift, Stellenbosch 7600, South Africa; 3 Department of Medical Sciences and Science for Life Laboratory, Uppsala University, SE-75237 Uppsala, Sweden; johanna.svensson@medsci.uu.se (J.S.); johanna.manninen@medsci.uu.se (J.M.); torbjorn.ost@medsci.uu.se (T.Ö.); jon.ramsell@medsci.uu.se (J.R.); matilda.aslin@medsci.uu.se (M.Å.); Tomas.Axelsson@medsci.uu.se (T.A.); Ulrika.Liljedahl@medsci.uu.se (U.L.); jessica.nordlund@medsci.uu.se (J.N.); Mikael.Aberg@medsci.uu.se (M.Å.); 4 Department of Women’s and Children’s Health, Uppsala University, SE-75185 Uppsala, Sweden; marta.lomnytska@akademiska.se (M.L.); karin.stalberg@kbh.uu.se (K.S.); 5 Department of Obstetrics and Gynaecology, Institute of Clinical Sciences, Sahlgrenska Academy at Gothenburg University, SE-41685 Gothenburg, Sweden; maria.lycke@vgregion.se (M.L.); karin.sundfeldt@obgyn.gu.se (K.S.); 6 Science for Life Laboratory, KTH-Royal Institute of Technology, SE-17165 Stockholm, Sweden; mathias.uhlen@scilifelab.se; 7 Swedish Collegium for Advanced Study, Thunbergsvägen 2, SE-752 38 Uppsala, Sweden

**Keywords:** ovarian cancer, protein biomarkers, early detection

## Abstract

**Simple Summary:**

Ovarian cancer is the eighth most common cancer among women and has a 5-year survival of only 30–50%. The survival is close to 90% for patients in stage I but only 20% for patients in stage IV. The presently available biomarkers have insufficient sensitivity and specificity for early detection and there is an urgent need to identify novel biomarkers. The aim of our study was to broadly measure protein biomarkers to find tests for the early detection of ovarian cancer. We found that combinations of 4–7 protein biomarkers can provide highly accurate detection of early- and late-stage ovarian cancer compared to benign conditions. The performance of the tests was then validated in a second independent cohort.

**Abstract:**

Background: Ovarian cancer is the eighth most common cancer among women and has a 5-year survival of only 30–50%. The survival is close to 90% for patients in stage I but only 20% for patients in stage IV. The presently available biomarkers have insufficient sensitivity and specificity for early detection and there is an urgent need to identify novel biomarkers. Methods: We employed the Explore PEA technology for high-precision analysis of 1463 plasma proteins and conducted a discovery and replication study using two clinical cohorts of previously untreated patients with benign or malignant ovarian tumours (*N* = 111 and *N* = 37). Results: The discovery analysis identified 32 proteins that had significantly higher levels in malignant cases as compared to benign diagnoses, and for 28 of these, the association was replicated in the second cohort. Multivariate modelling identified three highly accurate models based on 4 to 7 proteins each for separating benign tumours from early-stage and/or late-stage ovarian cancers, all with AUCs above 0.96 in the replication cohort. We also developed a model for separating the early-stage from the late-stage achieving an AUC of 0.81 in the replication cohort. These models were based on eleven proteins in total (ALPP, CXCL8, DPY30, IL6, IL12, KRT19, PAEP, TSPAN1, SIGLEC5, VTCN1, and WFDC2), notably without MUCIN-16. The majority of the associated proteins have been connected to ovarian cancer but not identified as potential biomarkers. Conclusions: The results show the ability of using high-precision proteomics for the identification of novel plasma protein biomarker candidates for the early detection of ovarian cancer.

## 1. Introduction

Early detection of ovarian cancer improves the 5-year survival rate, from close to 90% in stage I compared to only 20% when the cancer is discovered in stage IV [1]. Ovarian cancer is the eighth most common cancer among women today and kills over 200,000 women per year, worldwide [2]. Today, no molecular test that is accurate enough justify population-wide screening exists. The largest running ovarian cancer screening study, the United Kingdom Collaborative Trial of Ovarian Cancer Screening (UKCTOCS) [3], has evaluated multi-modal screening in post-menopausal women based on indications of elevated MUCIN-16 (Cancer antigen-125/CA125) followed by transvaginal ultrasound (TVU). A recent evaluation of the long-term results from the study [4] concluded that although a clear increase could be seen in early-stage cancer discovery, no clear improvement could be seen in reduced mortality rates in the screened group as compared to the un-screened group. These results have since been compared with the Normal Risk Ovarian Screening Study (NROSS) study which employed the same strategy but found a larger shift towards early discovery and more promising results [5]. One remaining issue with the multi-modal approach is the lower sensitivity of the biomarkers which are used, while a higher specificity is obtained with TVU [5]. Therefore, biomarkers with a higher sensitivity for early-stage detection are needed. At present, the discovery of ovarian cancer is usually symptom-driven, resulting in less than a third of the cases being discovered early, i.e., in stage I or II [1]. Women who experience pelvic symptoms are typically examined by available molecular biomarker analysis, TVU, or computer tomography, and when these indicate an adnexal ovarian mass, surgery is used for the final diagnosis. Today, in Sweden, close to four out of five women that undergo surgery for adnexal tumours are diagnosed with benign cysts, not cancer [6]. Access to more specific preoperative tools for separating benign and malignant conditions could therefore reduce unnecessary surgery and thus avoid associated complications and induced premature menopause. A few molecular biomarkers are clinically used today to complement imaging examinations, but none have sufficient accuracy to be used in the screening/early detection or for diagnostic purposes. MUCIN-16 was first suggested as a biomarker for ovarian cancer in 1983 [7] and is currently the best single biomarker used for diagnosis in post-menopause women and treatment management [8]. MUCIN-16, however, has a low sensitivity for early-stage cancer and a high rate of false positive indications in many benign gynaecological conditions in younger women, such as infections, pregnancies, and endometriosis [8]. MUCIN-16 has also been reported to be elevated above the clinically indicative cut-off for ovarian cancer (35 U/mL) in 5.1% of heart failures among elderly women [9] and in 45.7% of women with acute pancreatitis [10]. Additional biomarkers such as the WAP four-disulfide core domain 2 (WFDC2 or HE4) has been shown to increase the specificity in detection of ovarian cancer, especially in fertile women [11]. A combination of MUCIN-16 and WFDC2 is used in the ovarian malignancy risk algorithm (ROMA) index, which uses two different risk score calculations and cut-offs for pre- and post-menopausal women. The ROMA index, as originally described, achieved a sensitivity of 0.92 and a specificity of 0.75 in post-menopausal women, and a sensitivity of 0.77 and a specificity of 0.75 in pre-menopausal women [12]. A recent meta-analysis of the ROMA index in both pre- and post-menopausal women indicates that the overall sensitivity is in the range of 0.88 to 0.93 and the specificity is between 0.89 and 0.94 [13]. Apart from MUCIN-16 and WFDC2, which are used in the ROMA index, studies have indicated that additional protein biomarkers can be informative, e.g., for early diagnosis or screening. Russel and colleagues [14] combined MUCIN-16, Vitamin K-dependent protein Z (PROZ), phosphatidylcholine-sterol acyltransferase (LCAT) and C-reactive protein (CRP), into a multiplex biomarker panel that, when compared with a patient’s own baseline, showed promising performance in detecting ovarian cancer as early as 1–2 years prior to current diagnostic methods. We have previously presented a risk score based on 11 proteins that separate benign conditions from ovarian cancer (stage I–IV) at a sensitivity of 0.85 and a specificity of 0.93, regardless of menopausal state [15]. For early stages (stage I–II), the model achieved a sensitivity of 0.68 at a specificity of 0.93, compared to an optimized model based on MUCIN-16 and WFDC2, which reached a sensitivity of 0.56 and a specificity of 0.98 in the same set of samples [15]. Our model with 11 proteins included both MUCIN-16 and WFDC2 and was selected from a larger set of 593 proteins using the proximity extension assay (PEA). Our previous results motivate a broader search for novel biomarkers, using new technologies for large-scale plasma protein analyses. Our results also indicate that the proteins considered should not be restricted by prior knowledge of their involvement in ovarian cancer, nor by the need to attain univariate significance in order to be included in the multivariate modelling.

With this in mind, we have now used the novel PEA Explore technology to measure levels of 1472 proteins in plasma from two clinical cohorts (*N* = 111 and *N* = 37) and conducted both univariate and multivariate analyses to identify biomarker candidates for ovarian cancer.

## 2. Materials and Methods

### 2.1. Samples

Plasma samples of women with benign and malignant ovarian tumours were collected from the U-CAN collection [16] at Uppsala Biobank, Uppsala University, Sweden and the Gynaecology tumour biobank [17] at Biobank West, Sahlgrenska University Hospital, Göteborg, Sweden. All samples from the biobanks were included based on either primary ovarian cancer diagnosis or suspicion of ovarian cancer diagnosis but surgically diagnosed benign condition. Samples from patients that had begun neoadjuvant treatment prior to surgery were excluded. The samples from U-CAN were collected between 2012 and 2018 and the samples in the gynaecology tumour biobank were collected from 2016 to 2018. All tumours were examined by a pathologist specializing in gynaecologic cancers for histology, grade, and stage, according to the International Federation of Gynaecology and Obstetrics (FIGO) standards. All plasma samples were frozen and stored at −80 °C. In total, 148 unique patient samples were analyzed with 75% in the discovery cohort (111 patient samples from the Göteborg collection) and 25% in the replication cohort (37 patient samples from the Uppsala U-CAN collection). In the discovery cohort, 2/3 malignant and 1/3 benign samples were randomly selected from all available samples in the cohort. The samples in the replication cohort were randomly selected though preserving the malign/benign fraction and age distribution in the discovery cohort. Since the distribution of individual protein abundance levels in the samples was unknown, no univariate power calculations were carried out. Basic statistics for the samples used are presented in Table 1. Among the Göteborg collection, the benign histologies were, ordered by frequency: stromal (29.3%), mucinous (29.3%), serous (14.6%), teratoma (12.2%), simple (7.3%), endometriosis (4.9%) and myoma (2.4%). In the malignant samples, 75.7% were high-grade serous carcinoma (HGSC), 9.5% low-grade serous carcinoma (LGSC), 9.5% mucinous, 2.7% endometroid, and 2.7% clear cell cancer. In the U-CAN collection, the benign histologies were stromal (28.6%), mucinous (21.4%), endometriosis (21.4%), teratoma (14.3%), myoma (7.1%), and simple (7.1%). In the malignant samples, 52.2% were HGS, 21.7% LGS, 4.3% mucinous, 4.3% clear cell, and 4.3% were carcinosarcomas. Written consent was obtained from all participants before the samples used here were deposited in the biobanks. The study was approved by the Regional Ethics Committee in Uppsala (Dnr: 2016/145) and Göteborg (Dnr: 201-15).

### 2.2. Protein Measurements

The samples were analysed using the Olink Proteomics proximity extension assay (PEA) [18] Explore [19] at the SciLifeLab Explore Lab in Uppsala, Sweden. The samples were analyzed in two separate runs, where four of the benign samples from the Göteborg cohort were analysed in quadruplets in each. The detailed protocol for PEA Explore has been detailed previously by Wik and colleagues [19]. In brief, Olink’s PEA Explore technology is based on pairs of antibodies equipped with single-stranded DNA oligonucleotide reporter molecules, probes, that bind to their respective target if present in the sample. Target binding by both probes in a pair in close proximity generates double-stranded DNA amplicons. The PEA Explore assay is built upon four separate 384-plex panels focussing on inflammation, oncology, cardiometabolic and neurology proteins, corresponding to a total of 1472 human proteins. Three proteins (CXCL8, IL-6 and TNF) were included on each of the four separate 384-plex panels. After the initial probe-based immune reaction step in the PEA Explore workflow, the amplicons were extended and amplified in a two-step process where individual sample index sequences were added during the second step. After sample pooling, the libraries were prepared and sequenced on a NovaSeq 6000 instrument (Illumina, San Diego, CA, USA). The raw BCL files were transformed into count files, which in turn were translated into normalized protein expression (NPX) values through a quality control (QC) and normalization process built around internal and external controls, as specified by the manufacturer. The NPX data are on a log2 scale and in the logarithmic phase of the curve, one increase of the NPX value corresponds to a doubling of the protein content. A high NPX value corresponds to a high protein concentration. Each of the measured proteins has a lower limit of detection (LOD) which is determined at run time. The initial QC performed by the manufacturers’ proprietary analysis labels each specific assay and individual measurement with a warning. Here, nine specific protein assays were labelled with a warning and excluded from the analysis (Cardiometabolic panel, IL-19 and CST3; Neurology, SERPINB1 and MPO; Inflammation, CXCL8 and MMP1; Oncology, LYAR, KLK14 and DEFB4A/DEFB4B). Note that CXCL8 passed QC on the remaining three sub-panels and these measurements were kept. On the two plates, 7128 and 9696 corresponding to 5.5% and 7.5% of individual measurements were labelled with a QC warning and excluded from the analysis. No replacement or filtering of measurements under the specified limits of detection was done. On the two plates, 11,253 and 6482 of the individual measurements passing QC, corresponding to 8.7% and 5.0% of the total measurements, respectively, were under the LOD. After quality control, per-protein inter-plate normalization was carried out by adjusting the measurements with the mean difference of the 16 (four samples in quadruplets on each plate) biological and technical replicates. After the inter-plate normalization, one of the technical replicates was kept and the data from the two plates was merged. The final dataset consisted of 1463 proteins in 148 samples.

### 2.3. Statistical Analysis

All calculation were carried out using R [20] (version 4.0.3). Pairwise inter- and intra- plate-correlations were calculated with Pearson’s method using the ‘cor.test’-function. Beeswarm-plots were made using the R-package ‘beeswarm’ (0.4.0) [21]. Functional enrichment analysis was done using the DAVID tool [22] based on UniProt identifiers. Overlaps with TCGA [23] were studied based on the lists with UniProt ids available from the Human Protein Atlas (HPA) [24,25]. The original TCGA lists consists of genes with elevated expression that were associated with favourable/unfavourable outcomes and is provided as curated lists of UniProt ids at HPA. The literature search was conducted by searching PUBMED (https://pubmed.ncbi.nlm.nih.gov/ (accessed on the 6 December 2021)) for ‘ovarian cancer’ and (i) the protein short-name, (ii) the protein full name, and (iii) the name of the encoding gene. The multivariate models were selected and trained using the ‘cv.glmnet’ function from the ‘glmnet’ R-package (version 4.1.2) [26] using a three-fold cross validation, alpha set to 1, optimizing lambda in the range of 0.001 to 1 in steps of 0.001, and evaluated using receiver operating characteristics (ROC) and the area under the curve (AUC) as final error measure. The training data were first pruned to contain only non-missing observations across the protein, by stepwise removal of either individuals or proteins (highest proportion of missing values) until no missing values remained. The first set of models predicting separate cancer stages from benign conditions were trained starting from the set of proteins with univariate nominal significance as evaluated in the discovery set while the two disjunct models were trained using all proteins (first of the two) and then all except those in the first model. For all trained models, the final model coefficients were extracted from the cv-stage at a λ within one standard error of the minimum (‘lambda.1se’ in ‘cv.glmnet’). Outputs from the resulting linear model were transformed to a risk score in 0 to 1 using a link function: $f\left(x\right)=e^{x}/(e^{x}+1)$. After the models were trained, both the discovery and replication datasets were re-pruned to contain only non-missing values but considering only proteins present in a specific model. Performance of the model was then evaluated in the discovery and replication data. No samples from the replication data were used in training or optimization of the models. ROC plots, point estimates, confidence intervals of AUC, sensitivities, specificities, and cut-off for malignancy were calculated using the R-package ‘pROC’ (version 1.18.0) [27].

## 3. Results

### 3.1. Protein Measurements

One hundred and forty-eight (148) plasma samples from two separate clinical cohorts with women diagnosed with benign or malignant ovarian tumours were analysed using the PEA [18] Explore [19] technology. The first set of samples (*N* = 111, Table 1, discovery cohort) were from a cohort collected in Göteborg, Sweden and the second (*N* = 37, Table 1, replication cohort) were from the U-CAN biobank in Uppsala, Sweden (see Methods for details, including the distribution of histologies). The samples were analysed across two plates and four samples from the discovery cohort were analysed in quadruplets on both. From these four samples we calculated intra- and inter-plate correlations. All correlation estimates were found to be significant (*p* < machine precision (2.2 × 10^−16^)), with intra-plate correlations ranging from 0.94 to 0.99 and inter-plate correlations ranging from 0.87 to 0.95 (Supplementary Figure S1). After quality control and plate normalization (Methods), the final dataset consisted of 1463 proteins measured in 148 individuals.

### 3.2. Replicated Single Valued Biomarkers for Ovarian Cancer

**Figure 1:**
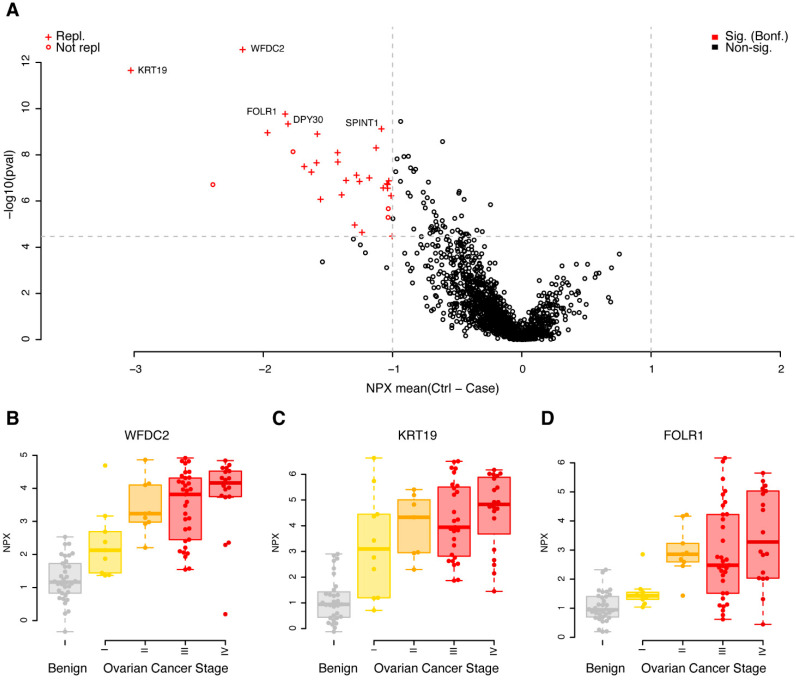
Univariate results in the discovery cohort. (**A**) Mean differences in NPX (controls–cases) are shown on the *x*-axis and *p*-values (−log10, two-sided Wilcoxon ranked test) on the *y*-axis. Proteins plotted in red were significantly different in the discovery data (q < 0.05, Bonferroni adjusted, and a foldchange of at least 1 NPX). Light grey dashed lines represent cut-offs for *p*-value and foldchange. Proteins marked with a ‘+’ were also found to be significant (q < 0.05, Bonferroni adjusted, foldchange of at least 1 NPX) in the replication data. The five proteins with the lowest *p*-values in the discovery data are labelled. (**B**) Beeswarm plots of individual protein measurements for WFDC2 in the discovery cohort. The top and bottom of the overlayed boxplots represents the 25th and 75th percentile and the band inside the box the median value. Outliers were omitted from the boxplots. The samples are divided by diagnoses: B—benign (coloured grey), I, II, III and IV—ovarian cancer FIGO stage (coloured yellow to red). (**C**) As (**B**), but for KRT19. (**D**) As (**B**), but for FOLR1.

A univariate case/control analysis was carried out comparing the protein concentrations between benign diagnoses with malignant, independent of cancer stage. Using strict adjustment for multiple hypothesis testing (Wilcoxon ranked test, *p* < 0.05/1463 = 3.4 × 10^−5^) and a minimum mean unsigned NPX difference between cases and controls of 1, corresponding to half/double the concentration on the log2 scale, we found 32 proteins that fulfilled these requirements in the discovery cohort (Figure 1A, Supplementary Table S1). All 32 proteins had higher concentrations in cases, when compared to controls (Figure 1A, Supplementary Table S1). The 32 proteins were brought forward to a replication phase where the same requirements on absolute difference in mean concentration and multiple hypothesis adjustment of statistical significance were used (*p* < 0.05/32 = 1.5 × 10^−3^). Using these settings, 87.5% (28 of 32) of the biomarkers were replicated (Figure 1A, Supplementary Table S1). The four non-replicated proteins were all nominally significant in the replication data, with *p*-values ranging from 1.5 × 10^−2^ to 2.9 × 10^−3^, with the same direction of change in cases compared to controls (the mean differences ranged from 0.90 to 2.0). Individual protein levels stratified on cancer stage for the top three proteins are shown in Figure 1B–D. In each of the three illustrated cases, a trend was observed between higher concentrations of the plasma proteins with higher cancer stage.

The PEA Explore technology broadly groups the proteins based on previously assigned function. Out of the 32 proteins found to be significant in the discovery cohort, 15 belonged to the Oncology panel, including the clinically used biomarkers WFDC2 and MUCIN-16, eight were found on the inflammation panel, seven on the neurology panel and two on the cardiometabolic panel. We further investigated if there was any enrichment of functional annotation, such as molecular function and biological process, among the significant proteins in the discovery analysis, using the gene ontology (GO) and enriched pathways such as KEGG. Using the complete set of the 1463 proteins analysed, we found no statistical enrichment of neither GO terms nor pathways in the group of 32 proteins, as compared to the background. This did not change when the entire human genome was used as the background. We next investigated the overlap with genes where their elevated expression is associated either favourably or unfavourably to ovarian cancer survival, using the results from the Cancer Genome Atlas (TCGA). Among the 1463 proteins, 41 overlapped in total, and in our list of 32 proteins, one protein (CXCL9) was found to overlap with the TCGA list. Given the total number of overlapping genes in the assay and the complete TCGA list, this is not a significant overlap (*p* = 0.60, binomial test). We found no trend relating the division of higher gene expression in tumour tissue being favourable/unfavourable (as reported by TCGA) with higher concentrations of circulating plasma proteins, as studied here (Supplementary Figure S2). A brief literature search (Methods), however, identified published connections on either genetic, transcriptional or protein levels with ovarian cancer in 26 of the 32 proteins (Supplementary Table S2).

### 3.3. New Multivariate Models Outperform Previous Biomarkers

**Figure 2:**
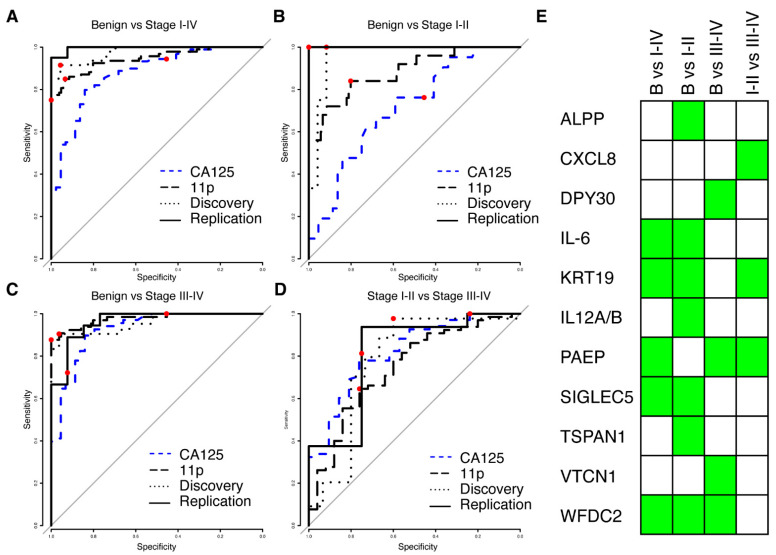
Results from multivariate modelling using the univariate significant biomarkers. (**A**) ROC curve from validation (solid line) and training (dotted line) for separating benign controls from ovarian cancer stages I–IV. The combined performance of clinically measured MUCIN-16 (CA125) in the same cohorts is shown in dashed blue and the performance of the previously developed 11-biomarker panel [15] is shown as a long-dashed black line. In each ROC curve, the point estimate of sensitivity and specificity at a cut-off (‘best-point’ for multivariate models, 35 U/mL for clinical MUCIN-16) is indicated by a red dot. (**B**,**C**) As (**A**), but for separating benign controls from stages I–II (**B**) and stages III–IV (**C**). (**D**) As (**A**), but for separating ovarian cancer stages I–II from stages III–IV. (**E**) Graphical illustration of the proteins (rows) included in each of the models in **A**–**D** (columns). A green box indicates inclusion.

We next proceeded to build multivariate predictive models separating benign from malignant tumours, starting from proteins (*N* = 175) with a nominal significance (*p* < 0.05, Wilcoxon ranked test) and a minimum mean NPX difference of 0.5 between the cases and controls in the discovery cohort. Four multivariate models were built: benign vs. stage I–IV, benign vs. stage I–II, benign vs. stage III–IV and finally stage I–II vs. stage III–IV. Each model was trained using the discovery cohort only and evaluated using the replication data. The model reports a risk score in the range of 0 to 1, and ROC (receiver operating characteristics) for all models in the replication and discovery cohorts are shown in Figure 2A–D. All models differentiating between benign and malignant conditions had an AUC of at least 0.96 in both discovery and replication, while the model separating early stages (I–II) from late stages (III–IV) achieved an AUC of 0.78 and 0.81 in the two cohorts (Table 2). None of the models showed a statistical difference between the discovery and replication cohorts (all *p*-values > 0.17, DeLong’s test, Table 2). For each of the models, a risk-score cut-off for malignancy was developed using the discovery cohort and defined as the point on the ROC curve closest to a perfect classification (best-point). We found no statistical difference in neither the achieved sensitivity nor specificity between the discovery and replication cohorts at the pre-defined cut-offs (Table 2), suggesting a robust performance of the models. The models consisted of three to seven proteins, with a total of eleven proteins across all models (Figure 2E). The model for early-stage detection (benign vs. I–II) consisted of seven proteins (ALPP, IL6, KRT19, P29460, SIGLEC5, TSPAN1, and WFDC2), the model for late-stage detection (benign vs. III–IV) of four proteins (DPY30, PAEP, VTCN1 and WFDC2), the model for all stages (benign vs. I–IV) of five proteins (IL6, KRT19, PAEP, SIGLEC5 and WFDC2) and the model separating early-stage from late stage (I–II vs. III–IV) consisted of three proteins (CXCL8, KRT19 and PAEP). None of the eleven proteins overlapped with the TCGA gene list for ovarian cancer. Two of the eleven proteins (KRT19 and WFDC2) overlapped with our previously published 11 biomarker panel for ovarian cancer [15]. Our multivariate models showed consistently higher AUC for all comparisons between benign and malignant samples in comparison to clinically measured MUCIN-16 in the patients in our two cohorts (Figure 2A–C, Supplementary Table S3). In comparison to clinically measured MUCIN-16, we specifically found statistically significant higher AUCs (Bonferroni adjusted q < 0.05, DeLong’s test) in both cohorts when comparing the benign to the early stages (stage I and II) and when comparing benign to all stages (Stage I–IV). There was, however, no statistically significant difference when comparing benign to late-stage cancers (stage II–IV, all *p* < 0.33).

We also compared the performance of the multivariate models developed here with our previously described 11-protein biomarker model [15] and found that the new models had higher AUCs when comparing benign to both early stage (I–II) and all stages (stage I–IV). This difference was statistically significant in the replication cohort (Bonferroni adjusted q < 0.05, DeLong’s test) but not in the discovery cohort. In the comparison between benign and late-stage (stages III–IV), our previous 11-protein biomarker model showed higher AUCs when compared to the multivariate models developed here (0.977 vs. 0.962 and 0.958 in the discovery and replication cohort, respectively), However, this difference was not statistically significant (Figure 2C, all *p* > 0.42, DeLong’s test). There was also no statistical difference in the AUC estimates when separating late-stage (III–IV) from early stage (I–II) cancer (Figure 2D, all *p* > 0.68, DeLong’s test). Lastly, we compared the sensitivity and specificity in the replication cohort at the ‘best-point’ cut-off with a similarly defined cut-off in our previously developed 11-protein biomarker model [15]. The new model showed higher sensitivity and specificity for early detection (stage I–II) and overall detection (stage I–IV), but lower sensitivity and specificity for late-stage detection (stage III–IV). None of these differences were statistically significant (Figure 2A–C, all *p* > 0.11, Fisher’s exact test). All point estimates of performance measures and statistical evaluations are given in Supplementary Table S3.

### 3.4. High Performance Models Are Not Unique

**Figure 3:**
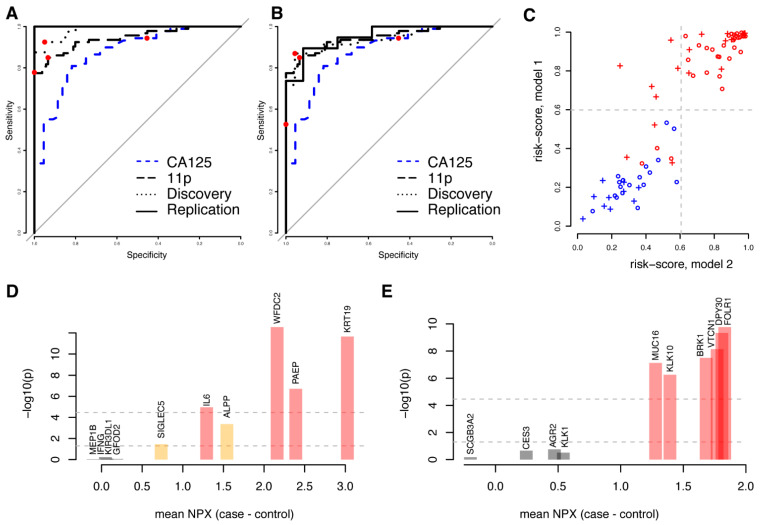
Comparison of two disjunct multivariate models based on all proteins. (**A**) ROC curve for the first model (‘m1′, including ALPP, GFOD2, IFNG, IL6, KIR3DL1, KRT19, MEP1B, PAEP, SIGLEC5, and WFDC2) in the validation (solid line) and training (dashed line) cohorts for separating benign controls from ovarian cancer stages I–IV. The combined performance of clinically measured MUCIN-16 (CA125) in the same cohorts is shown as a dashed blue line and the performance of the previously developed eleven-biomarker panel [15] is shown as a long-dashed black line. In each ROC curve, the point estimate of sensitivity and specificity at a cut-off (‘best-point’ for multivariate models, 35 U/mL for clinical MUCIN-16) is indicated by a red dot. (**B**) As (**A**), but for the second model (‘m2′, including AGR2, BRK1, CES3, DPY30, FOLR1, KLK1, KLK10, MUCIN-16, SCGB3A2, and VTCN1). (**C**) Comparison of risk scores for each individual in the training cohort (circles) and validation cohort (crosses) for the two models. Benign samples are illustrated in blue and malignant samples are illustrated in red. The dashed light-grey lines correspond to the cut-off for malignancy in the two models: 0.599 for the first model (*y*-axis) and 0.606 for the second (*x*-axis). (**D**) Univariate results for the proteins in the first model. The *x*-axis indicates mean differences between cases and controls in the discovery cohort and the *y*-axis represents the statistical significance of this difference (in −log10). The two horizontal lines indicate nominal significance levels and those adjusted for multiple hypothesis testing and is also indicated by the colours of the bars. (**E**) As (**D**), but for the second model.

We reasoned that not only proteins showing univariate significance could be predictive in multivariate models and subsequently built a model predicting malignancy (benign vs. stage I–IV) using the complete set of 1463 proteins studied. Using the same framework as above (Methods), a first model (m1) consisting of ten proteins (ALPP, GFOD2, IFNG, IL6, KIR3DL1, KRT19, MEP1B, PAEP, SIGLEC5, and WFDC2) was developed. The model achieved an AUC of 0.98 with the discovery data and 1.00 with the replication data (Figure 3A, Table 3). Similar to the models based on univariately significant proteins, there was no statistical difference in neither the sensitivity nor specificity between the cohorts at the pre-defined cut-off developed in the discovery cohort (Table 3). We then repeated the model-generating process excluding the ten proteins included in the first model (m1) from the possible selections and built a second (m2) model predicting malignancy (benign vs. stage I–IV). The second model (m2), with FOLR1, KLK10, KLK1, DPY30, MUCIN-16, CES3, VTCN1, SCGB3A2, AGR2 and BRK1, achieved an AUC of 0.94 with the discovery data and 0.95 with the replication data (Figure 3B, Table 3). There was no statistically significant difference in the specificity between the cohorts at a pre-defined cut-off developed in the discovery cohort, although a significantly lower sensitivity was observed (*p* = 0.00073, Fisher’s exact test, Table 3). Even though the two models were based on entirely different sets of biomarkers, the individual risk scores were highly correlated both for the discovery (Figure 3C, Spearman’s Rho = 0.90, *p* < machine precision = 2.2 × 10^−16^) and the replication cohorts (Spearman’s Rho = 0.87, *p* < 1.4 × 10^−6^). As above, we also compared the performance of the two models generated from the full protein dataset with our previously (Figure 3C) developed eleven-biomarker model [15] and with clinically measured MUCIN-16 (Figure 3A,B) in the patient samples analysed here. Based on the replication cohort, we found that the first ten-protein model (m1) had a statistically higher AUC (all *p* < 7.6 × 10^−4^, DeLong’s test, Supplementary Table S3) than the previous eleven-biomarker model [15]. The second model (m2) also showed a higher AUC than the eleven-biomarker model, although the differences were not statistically significant (all *p* > 0.85, DeLong’s test, Supplementary Table S3). At the ‘best-point’ cut-off, both new models showed a non-significant higher specificity (Figure 3A,B, *p* = 1.0, Fisher’s exact test, Supplementary Table S3), as compared to the previously developed eleven-biomarker model. The first model (m1) had a non-significant lower sensitivity (*p* = 0.49, Fisher’s exact test, Supplementary Table S3), while a significantly lower sensitivity (*p* = 3.6 × 10^−3^, Fisher’s exact test, Supplementary Table S3) was observed for the second model (m2). There was no statistical difference in neither AUC, sensitivity, nor specificity when comparing the two models to each other (Supplementary Table S3).

Four of the proteins (IL-6, KRT19, PAEP, and WFDC2) in the first model (m1) were present in the 32 proteins that showed univariate significance after multiple hypotheses adjustment, and an additional two (ALPP and SIGLEC5) were among the 175 proteins that were nominally significant (Figure 3D). In the second model (m2), six proteins (BRK1, DPY30, FOLR1, KLK10, MUCIN-16, and VTCN1) overlapped with the 32 proteins with univariate significance (Figure 3E). No additional proteins overlapped with the 175 nominally significant proteins. None of the proteins in either model overlapped with the TCGA gene lists for ovarian cancer. Both models each had two proteins (KRT19, WFDC2 and FOL1R, MUCIN-16, respectively) overlapping with our previously published 11-biomarker panel for ovarian cancer [15].

## 4. Discussion

We investigated a large set of proteins for their ability to identify women with ovarian cancer and found novel combinations of biomarkers that outperform both our previous biomarker panel [15] and clinically measured MUCIN-16 in the cohorts used here. We also showed that it is possible to define multiple, completely disjointed models, that have similar performances in terms of separating benign and malignant conditions. It is likely that future studies with additional proteins could identify similar or better performing multivariate models for ovarian cancer prediction. There is, however, a strong need to identify biomarkers that can aid in the early detection of ovarian cancer to increase survival. Today, none of the available biomarkers are accurate enough to identify early-stage ovarian cancers (high sensitivity) without also including a considerable fraction of false positives (low specificity). False positives lead to unnecessary anxiety in women until a benign condition has been confirmed; it also leads to costly examinations, additional burden to the health care system, unnecessary surgery, and risks of increased morbidity and infertility. Moreover, preoperative rupture of ovarian cancer decreases patients’ survival; thus, diagnostic biopsies of suspected ovarian cancer adnexal lesions should be avoided. A biomarker, single-valued or multivariate, can be tuned by choosing the most suitable cut-off to prioritize between sensitivity and specificity. For a given model, increased sensitivity comes at the cost of lowering specificity. Apart from TVU, molecular biomarkers, such as the analysis of circulating tumour DNA (ctDNA), have been shown to have very high specificity for cancer detection in general, particularly ovarian cancer [28,29]. A test based on multiple types of biomarkers could potentially be developed that shows both high sensitivity and specificity. The interval of testing in relation to sensitivity and specificity has also been evaluated by previous studies and intervals as short as three [30] and four [31] months have been suggested to improve the accuracy of MUCIN-16 by comparing the individuals’ own baseline over time. Although shorter intervals of testing provide a good basis for the early detection of changes in an individuals’ baseline, such strategies put high demands on health providers, individual long-term compliance, and the cost and throughput requirements of the tests used.

The majority of the proteins showing univariate differences between cases and controls in our analyses have previously been connected to ovarian cancer in the literature, either in terms of protein levels or gene expression levels, based on either cell line studies or patient cohorts. In contrast, and in accordance with our previous study of plasma protein biomarkers for ovarian cancer [15], many of the proteins included in the multivariate models do not reach univariate significance when adjusted for multiple hypothesis testing, nor have they been clearly connected to ovarian cancer in the existing literature. In our first set of four models for separating benign tumours from ovarian cancer (all stages, early stages or late stages) and early stage from late stage, a total of eleven proteins was included. A first selection was performed based on univariate nominal significance and of the eleven proteins, six (DPY30, IL6, KRT19, PAEP, VTCN1, and WFDC2) were among the 32 also showing significance after multiple hypothesis adjustment, but five (ALPP, CXCL8, IL12A/B, TSPAN1, and SIGLEC5) did not. Apart from the well-documented WCDF2, which is a clinically used biomarker for ovarian cancer today, several of the ten proteins in the multivariate model have been specifically associated with ovarian cancer in the literature. VTCN1 (V-set domain containing T cell activation inhibitor 1), has been suggested as a possible target for treatment, as its overexpression has been linked to viability and metastasis in ovarian cancer [32]. Higher expression of glycodelin A (PAEP) in ovarian cancer tumours is associated with both a lower overall patient survival and a shorter relapse-free survival time [33]. A recent study reported higher plasma levels of interleukin 6 (IL6) in patients with high-grade serous ovarian cancer compared to benign tumours and healthy controls [34]. DPY30 is suggested as an oncogene for ovarian cancer and high expression of DPY30 is associated with reduced survival in patients [35]. Experimental evidence suggests that a higher expression of CXCL8 in ovarian cancer cells is associated with metastasis and angiogenesis [36,37]. TSPAN1 is reported to be overexpressed in both ovarian cancer tissue and cell lines [38]. Keratin 19 (KRT19) is reported to be upregulated in response to the overexpression of the kallikrein related peptidase 4 (KLK4), with suggested a downstream increase of malignancy by lower sensitivity to paclitaxel treatment in ovarian cancer [39]. Both proteins were upregulated and nominally statistically significant (Supplementary Table S1) in both our cohorts, but only the difference of KRT19 remained significant after adjustment for multiple hypothesis testing. The expression pattern in tumour tissue of the glycosylphosphatidylinositol (GPI)-anchored placental alkaline phosphatase (ALPP) is suggested as a complementary biomarker to MUCIN-16 and WFDC2 for early detection in serous ovarian carcinoma [40]. We found no direct link between IL12A/B with ovarian cancer, although pre-activation with IL-12 in ovarian cancer cell lines is shown to improve the efficacy of natural killer cell immunotherapy [41]. Finally, SIGLEC5 is suggested as a prognostic marker for colorectal cancer [42] but we found no previously published association with ovarian cancer.

The strengths of our study are that we analysed a large set of proteins with and without prior association to ovarian cancer in two separate cohorts. The two cohorts were used strictly as discovery and replication cohorts both for the univariate and multivariate analyses. Both included cohorts contained a distribution of histology diagnoses both among the malignant and benign samples and in both the univariate and multivariate analyses, the obtained results were largely replicated. Our study is limited by the sample size, firstly by the size of the replication cohort, which may not be large enough to enable statistical significance in the replication steps, and secondly by restricting us from stratified analyses of different histologies or stages. The current study also only included samples collected at the time of diagnosis, and we are unable to analyse when the proposed biomarkers models would signal malignancy, in relation to diagnose by current methods.. Compared with the highly expressed genes associated with either favourable or unfavourable outcomes as indicated by the TCGA, we found a small overall overlap with the proteins studied here. There was also no clear relationship between elevated protein expression and classification as favourable or unfavourable for cancer development. However, TCGA is based on analysis of gene expression in biopsies from high-grade serous cancer only, while our study is based on circulating protein levels in plasma from a distribution of ovarian tumour histologies.

## 5. Conclusions

Recent advances in the throughput of ultra-high sensitivity proteomics technologies, such as PEA Explore, have enabled characterization of an increasingly large fraction of the plasma proteome using very small quantities of input material. Coupling such sensitive analysis technologies with machine learning approaches to detect combinations of biomarkers with robust predictive power, is a powerful approach to break new ground and enable progress beyond the current knowledge. The PEA Explore technology can be applied not only to liquid plasma samples, but also to other clinical sample matrices, such as dried blood spots [43,44]. This opens the possibility to establish screening programs based on self-collected clinical samples, coupled with highly sensitive analysis of precise molecular biomarkers, as a cost-efficient solution for the early detection of ovarian cancer.

## Figures and Tables

**Table 1:** Cohort characteristics.

	Cohort	All	Benign	Ovarian Cancer			
				I	II	III	IV
No. of samples	Discovery	111	37	10	9	35	20
	Replication	37	14	4	0	10	9
Age at diag. ^a^	Discovery	60.1 (13.2)	56.2 (15.1)	62.0 (12.2)	66.4 (6.2)	60.6 (12.5)	62.4 (12.4)
	Replication	57.4 (14.4)	49.9 (14.9)	70.4 (15.6)		60.2 (6.8)	60.3 (14.9)
Age diff *p*-value ^b^		0.21	0.11	0.36		0.52	0.83
CA125 (U/mL) ^c^	Discovery	263 (358.8)	41.5 (40)	67 (65.2)	240 (213.5)	594 (551.5)	1358 (1693.1)
	Replication	189 (255)	28.5 (24.5)	189 (235.7)		640 (763.5)	340 (315.8)
CA125 diff *p*-value ^b^		0.64	0.46	0.48		0.76	0.31

^a^ Reported as mean (standard deviation) age in the group. ^b^ Two-sided Wilcoxon ranked test of difference between the two cohorts. ^c^ Reported as median (median absolute deviation) CA125 in the group.

**Table 2:** Results of multivariate modelling based on proteins with nominal univariate significance.

		AUC ^a^	Sens ^b^	Spec ^b^
B vs. I–IV	Discovery	0.98 (0.95–1.00)	0.91 (0.83–0.98)	0.96 (0.87–1.00)
	Replication	1.00 (0.99–1.00)	0.75 (0.55–0.95)	1.00 (1.00–1.00)
	*p*-value ^c^	0.17	0.11	1.00
B vs. I–II	Discovery	0.96 (0.90–1.00)	1.00 (1.00–1.00)	0.92 (0.79–1.00)
	Replication	1.00 (1.00–1.00)	1.00 (1.00–1.00)	1.00 (1.00–1.00)
	*p*-value ^c^	0.22	1.00	0.53
B vs. III–IV	Discovery	0.96 (0.92–1.00)	0.90 (0.81–0.98)	0.96 (0.89–1.00)
	Replication	0.96 (0.90–1.00)	0.72 (0.50–0.89)	0.92 (0.77–1.00)
	*p*-value ^c^	0.92	0.11	1.00
I–II vs. III–IV	Discovery	0.78 (0.61–0.95)	0.98 (0.93–1.00)	0.60 (0.33–0.80)
	Replication	0.81 (0.51–1.00)	0.81 (0.62–1.00)	0.75 (0.25–1.00)
	*p*-value ^c^	0.85	0.054	1.00

^a^ Numbers for discovery and replication are given as a point estimate and 95% confidence intervals. ^b^ The point estimate and 95% confidence interval is given at a cut-off defined in the discovery cohort at the point on the ROC (receiver operating characteristics) curve closest to perfect classification. ^c^ *p*-values for difference in AUCs were calculated using the DeLong’s method. For differences of sensitivity and specificity, a Fisher’s exact test was used. B—Benign. Roman numerals specify ovarian cancer stage.

**Table 3:** Results from the multivariate modelling using all proteins.

		AUC ^a^	Sens ^b^	Spec ^b^
Model 1	Discovery	0.98 (0.96–1.00)	0.93 (0.82–1.00)	0.95 (0.85–1.00)
	Replication	1.00 (1.00–1.00)	0.78 (0.56–0.94)	1.00 (1.00–1.00)
	*p*-value ^c^	0.16	0.19	1.00
Model 2	Discovery	0.94 (0.89–0.99)	0.87 (0.76–0.96)	0.96 (0.87–1.00)
	Replication	0.95 (0.89–1.00)	0.53 (0.32–0.74)	1.00 (1.00–1.00)
	*p*-value ^c^	0.78	0.0073	1.00

^a^ Numbers for discovery and replication are given as point estimates and 95% confidence intervals. ^b^ The point estimate and 95% confidence interval is given at a cut-off defined in the discovery cohort at the point on the ROC (receiver operating characteristics) curve closest to perfect classification. ^c^ *p*-values for difference in AUCs was calculated using the DeLong’s method. For the differences of sensitivity and specificity, a Fisher’s exact test was used.

## Data Availability

The raw data, combined with the clinical data, are considered to be sensitive but can be made available through reasonable request.

## Supplementary Materials

The following supporting information can be downloaded at: https://www.mdpi.com/article/10.3390/cancers14071757/s1, Figure S1: Correlations in technical replicates; Figure S2: Univariate results of genes implicated as unfavourable or favourable in relation to high RNA expression in tumour tissue as reported in TCGA (The Cancer Genome Atlas); Table S1: Results from univariate analysis; Table S2: Previous literature connections; Table S3: Results from the multivariate modelling.

## Abbreviations

AGR2—Anterior gradient protein 2 homolog, ALPP—Alkaline phosphatase, placental type, BRK1—Protein BRICK1, CES3—Carboxylesterase 3, CST3—Cystatin-C, CXCL8—Interleukin-8, DEFB4A/B—Beta-defensin 4A/B, DPY30—Protein dpy-30 homolog, FOLR1—Folate receptor alpha, GFOD2—Glucose-fructose oxidoreductase domain-containing protein 2, IFNG—Interferon gamma, IL12—Interleukin 12, IL19—Interleukin-19, IL6—Interleukin-6, KIR3DL1—Killer cell immunoglobulin-like receptor 3DL1, KLK1—Kallikrein-1, KLK10—Kallikrein-10, KLK14—Kallikrein-14, KRT19—Keratin, type I cytoskeletal 19, LYAR—Cell growth-regulating nucleolar protein, MEP1B—Meprin A subunit beta, MMP1—Interstitial collagenase, MPO—Myeloperoxidase, MUC16—Mucin-16, PAEP—Glycodelin, SCGB3A2—Secretoglobin family 3A member 2, SERPINB1—Leukocyte elastase inhibitor, SIGLEC5—Sialic acid-binding Ig-like lectin 5, TNF—Tumor necrosis factor, TSPAN1—Tetraspanin-1, VTCN1—V-set domain-containing T-cell activation inhibitor 1, WFDC2—WAP four-disulfide core domain protein 2.
