# Supplementary material for: Next Generation Plasma Proteomics Identifies High-Precision Biomarker Candidates for Ovarian Cancer
Source: Cancers (Basel). 2022 Mar 30;14(7):1757. doi: 10.3390/cancers14071757 (PMC8997113; doi:10.3390/cancers14071757)
Supplement: Supplementary file 1 [file cancers-14-01757-s001.zip › Gyllensten_ocExplore_220121_suplFigures.pdf]

Supplementary Figures

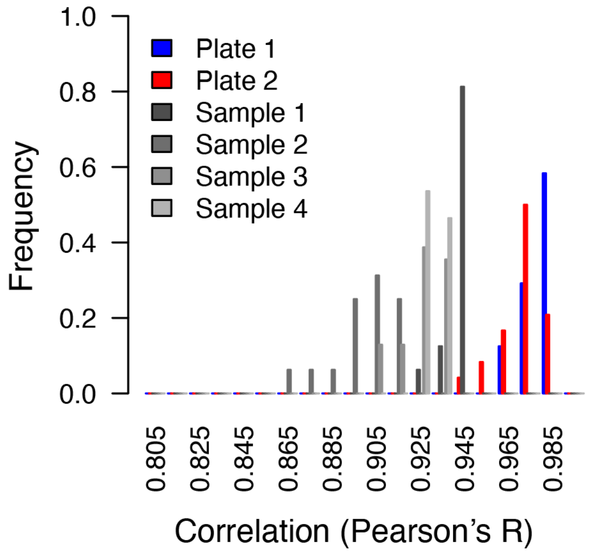

Supplementary Figure S1. Correlations in technical replicates. Distribution of within-plate correlation coefficients (Pearson's R) are shown in blue and red. The correlations are based on the four samples that were run in quadruplets on each plate, in total 48 comparisons (6 within-sample comparisons for four samples on two plates). Individual between-plates correlations (Pearson's R) for the four samples are shown in grey scale. In total 64 comparisons, each sample on one plate is compared to the 4 runs on the other (4x4x4). Numbers on the x-axis indicate the mean of the range in each range (+/- 0.005). X-axis has been truncated to 0.8 – 1.0.

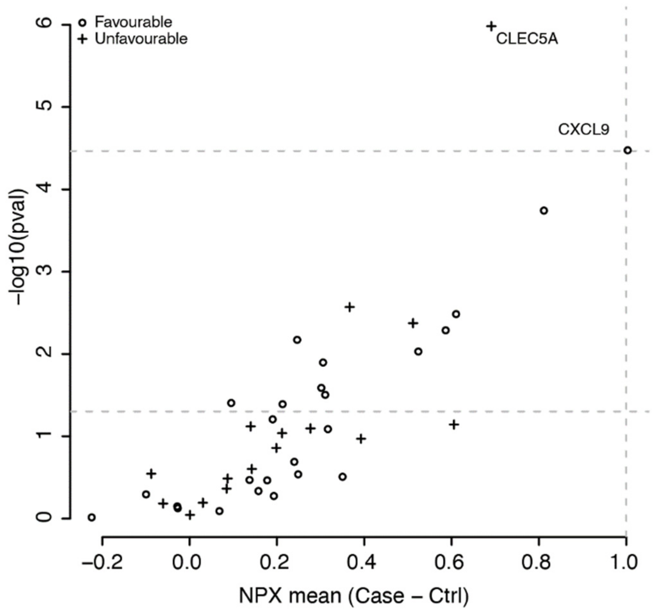

Supplementary Figure S2. Univariate results of genes implicated as unfavourable or favourable in relation to high RNA expression in tumour tissue as reported in TCGA (The Cancer Genome Atlas). Proteins mapping to favourable genes are indicated with a circle and

unfavourable with a cross. Y-axis shows -log10(p-value) of difference in plasma protein concentration between cases and controls. X-axis show mean difference between cases and controls in NPX. The horizontal lines represent a nominally statistical difference ($p < 0.05$, about 1.3 on the -log10 scale) and the Bonferroni adjusted cut-off used here ($p < 0.05/1463 = 3.4 \times 10^{-5}$, about 4.4 on the -log10 scale). The vertical line represents minimum difference of 1.0 NPX between cases and controls used here. Proteins with p-values below the Bonferroni adjusted cut-off are labelled in the figure. Only CXCL9 were among the proteins brought forward from the discovery stage.
